# Fertilization shapes a well-organized community of bacterial decomposers for accelerated paddy straw degradation

**DOI:** 10.1038/s41598-018-26375-8

**Published:** 2018-05-22

**Authors:** Yushan Zhan, Wenjing Liu, Yuanyuan Bao, Jianwei Zhang, Evangelos Petropoulos, Zhongpei Li, Xiangui Lin, Youzhi Feng

**Affiliations:** 1 State Key Laboratory of Soil and Sustainable Agriculture, Institute of Soil Science, Chinese Academy of Sciences, Nanjing, 210008 P.R. China; 2 School of Civil Engineering and Geosciences, Newcastle University, Newcastle upon Tyne, NE1 7RU UK

## Abstract

Straw, mainly dry stalks of crops, is an agricultural byproduct. Its incorporation to soils via microbial redistribution is an environment-friendly way to increase fertility. Fertilization influences soil microorganisms and straw degradation. However, our up to date knowledge on the responses of the straw decomposers to fertilization remains elusive. To this end, inoculated with paddy soils with 26-year applications of chemical fertilizers, organic amendments or controls without fertilization, microcosms were anoxically incubated with ^13^C-labelled rice straw amendment. DNA-based stable isotope probing and molecular ecological network analysis were conducted to unravel how straw degrading bacterial species shift in responses to fertilizations, as well as evaluate what their roles/links in the microbiome are. It was found that only a small percentage of the community ecotypes was participating into straw degradation under both fertilizations. Fertilization, especially with organic amendments decreased the predominance of *Firmicutes*- and *Acidobacteria*-like straw decomposers but increased those of the copiotrophs, such as β-*Proteobacteria* and *Bacteroidetes* due to increased soil fertility. For the same reason, fertilization shifted the hub species towards those of high degrading potential and created a more stable and efficient microbial consortium. These findings indicate that fertilization shapes a well-organized community of decomposers for accelerated straw degradation.

## Introduction

Rice production is expected to significantly increase in the near future to meet the demand of the rising human population. Nowadays, paddy rice culture produces 660 m tons of rice annually, generating approximately 800 m dry tons of agricultural waste, mainly straw^1^. China is one of the largest rice resources worldwide^2,3^. Its common traditional ‘waste management’ of produced rice straw includes burning on site. This results in the formation of hazardous waste for the public health, airborne emissions as well as large volumes of greenhouse gases^4^. Rice straw is one of the key organic carbon sources for arable soils improving soil’s physical and chemical quality via microbial redistribution^5^. It is illustrated that the main components of rice straw are hemicellulose (26–35%), cellulose (38–41%), lignin (15%), and water-soluble polysaccharides (8%)^6^. Due to the complex and recalcitrant nature of these compounds, a well-structured microbial community is required for successful bioconversion^7–10^. In parallel, the strong intra- and inter-species cooperation (such as synergy, syntrophy and/or symbiosis) is strictly required for sufficient straw decomposition^11–13^. Therefore, in the past decades, scientists have never stopped studying the community of straw decomposers.

Over the last decade, several multidisciplinary methods have been developed and applied for the links of the identities of straw decomposers with their activities and functions. Stable isotope probing (SIP) was proven to be a promising approach^14–17^. Murase *et al*.^18^, by incorporating ^13^C rice straw into submerged soils and via phospholipid fatty acid (PLFA)-SIP analysis, found that both Gram-negative and -positive bacteria are the key microbial members in the paddy straw degradation process. Similarly, Shrestha *et al*.^19^ used RNA-SIP via ^13^C-labeled rice straw and identified that members of the *Clostridium* cluster are actively involved in straw degradation in paddy soil. The importance of the *Clostridia* classes, followed by *Acidobacteria*, *Bacteroidetes* and *Proteobacteria* were also reported by Lee *et al*.^20^ who used DNA-SIP in anoxic rice callus treated microcosms. These community patterns deepened our understanding with regards the key species and the functions of the straw degrading microorganisms present in soils.

Fertilization is an important agricultural practice that aims to the improvement of plant nutrition to achieve high crop yield. It has been previously showed that fertilization is beneficial to straw degradation^21–24^. A possible explanation is that fertilization could alter the community composition of straw decomposers because of the response of cells to environmental changes. As clues, Eichorst and Kuske^25^ found that edaphic and geographic soil characteristics may alter the composition of the cellulose-responsive microbial community. At a local scale, Koranda *et al*.^24^ found that the enhanced N availability increases fungal degradation of cellulose. Apart from the community-level alterations, fertilization could also affect the biotic interaction of the consortia involved into decomposition. Recently, molecular ecological network analysis was widely used to understand the potential biotic interactions between habitat affinities and shared physiologies^26,27^. This new statistical approach offers novel insights into the understanding of the microbiome and the significance of specific members in the community^26,28^. Analysis of molecular ecological networks in soils fertilized organic amendments showed that microbial communities are more decentralized and assigned to more ecological modules in comparison with the communities in soils under chemical fertilization^29,30^. This implies that organic amendments can benefit soil microbiome with stronger ecological function potential and higher stability. The above information leads to our hypotheses that fertilization (i) promotes the development of an efficient rice straw decomposing community; and (ii) influences the synergistic interactions between consortia, both of which lead to enhanced and accelerated rice straw degradation.

To confirm the above hypotheses we employed high-abundance ^13^C-labeled rice straw by a labeling technique in which a rice-soil system was placed in a gas-tight growth chamber in an enriched of ^13^C-CO_2_ atmosphere. The paddy soils with three 26-year fertilization regimes (chemical fertilizers (termed NPK afterward), organic amendments (OM) or without fertilization (Control)) were collected to anoxically incubate microcosms in our laboratory. For each soil, incubations were carried out under three different straw amendment strategies: regular rice straw (^12^C-straw), ^13^C-labeled straw (^13^C-straw), and without straw (Unamended). Bioinformatics analysis on fractionated and non-fractionated DNAs was conducted to (i) identify the bacterial taxa associated with the assimilation of ^13^C derived from ^13^C-labled rice straw; (ii) unravel the influence of different fertilization regimes on the paddy straw degrading species and their hub roles in the associated molecular ecological networks.

## Results

### Microbial biogas evolution in microcosms

**Figure 1:**
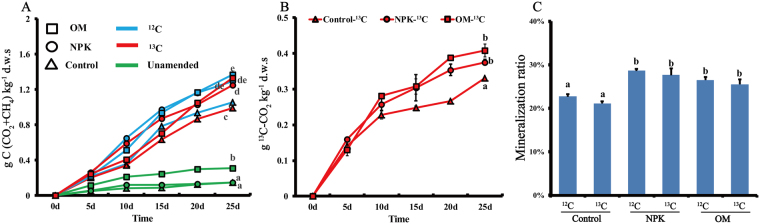
(**A**) Biogas (CO_2_ and CH_4_) and (**B**) ^13^C-CO_2_ production rates from the soil microcosms; (**C**) net C mineralization ratio over the total C from the amended straw. NPK, OM and Control denote the soils under 25-year chemical balanced fertilizers, organic amendments and without fertilization respectively. ^13^C and ^12^C denote the soils amended with ^13^C-straw and ^12^C-straw respectively. Unamended denotes treatments without straw addition. Square, circle and triangle indicate OM, NPK and Control soils. Blue, red and green lines represent ^12^C- and ^13^C-straw amendment and Unamended treatments. Standard error bars were obtained from 8 replicates. The different letters above error bars indicate significance.

Generally, biogas (CO_2_ + CH_4_) accumulations for the straw amendments (^12^C-straw treatments in Fig. 1A: 1.054 g kg^−1^ (Control), 1.290 g kg^−1^ (NPK) and 1.370 g kg^−1^ (OM) and ^13^C-straw treatments: 0.991, 1.251 and 1.332 g kg^−1^) were significantly higher than those without straw amendment (Unamended treatments: 0.147, 0.145 and 0.311 g kg^−1^) (*P* < 0.01). This implies that straw amendment stimulates the anoxic microbial metabolism. This is also supported by the evaluation of the ^13^C-CO_2_ emission data: comparing with the ^12^C-straw treatments (0.008 g kg^−1^ (Control), 0.009 g kg^−1^ (NPK) and 0.010 g kg^−1^ (OM)), the ^13^C-straw treatments had significantly higher cumulative ^13^C-CO_2_ emissions (0.330, 0.375 and 0.410 g kg^−1^) (Fig. 1B). Furthermore, after 25 days it was shown that fertilized soils had a higher net C mineralization ratio over the total C from the amended straw compared to those achieved by the Control soils (Fig. 1C).

### Quantitative analysis of bacterial 16S rRNA gene across isotopically fractionated DNA gradients

**Figure 2:**
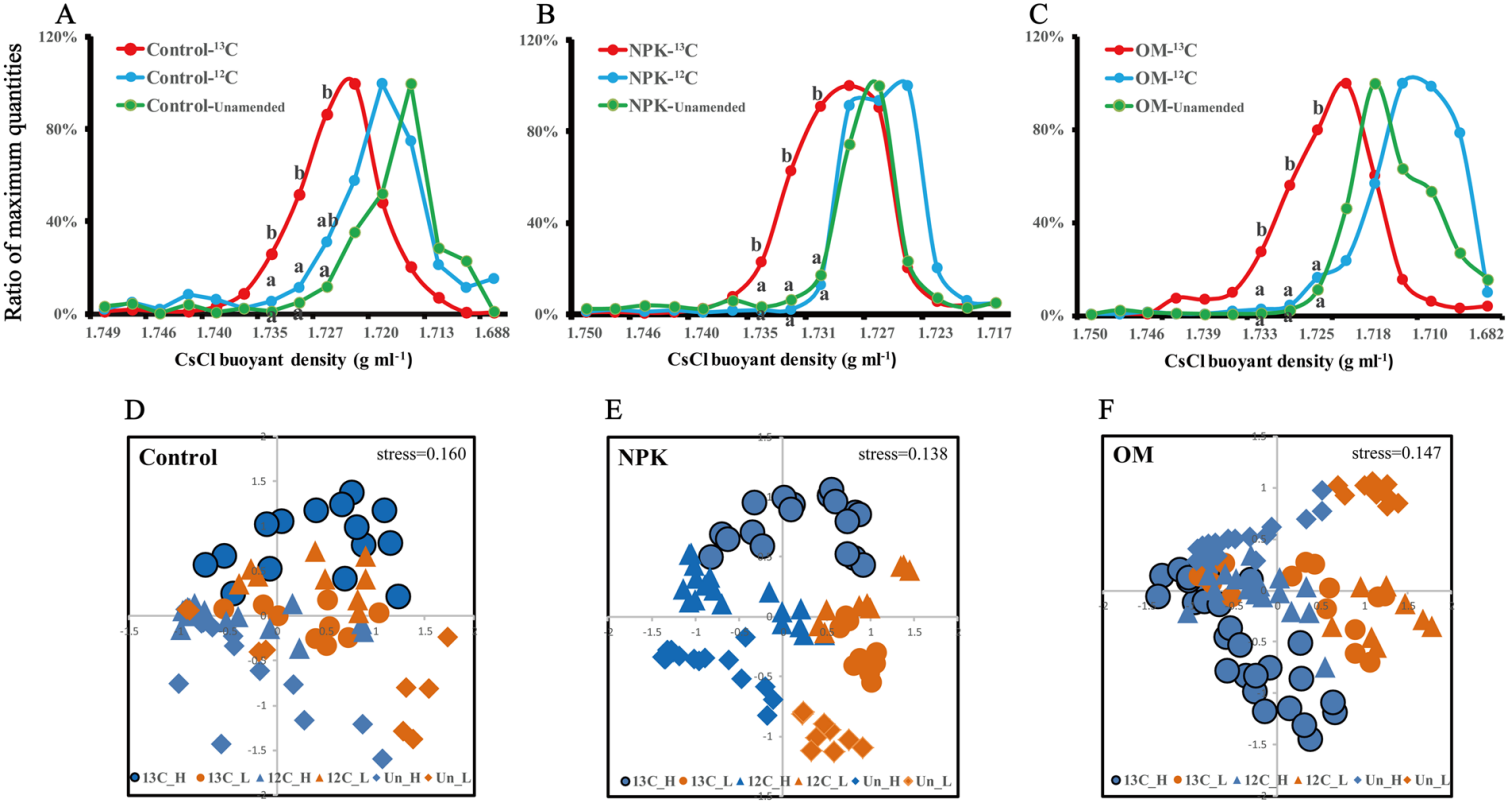
Quantitative distribution of bacterial 16S rRNA gene copy numbers across the entire buoyant densities of DNA gradients isolated from Control (**A**), NPK (**B**) and OM (**C**) soils incubated with ^13^C-straw, ^12^C-straw or Unamended respectively. The normalized data are the ratio of the gene copy number in each DNA gradient fraction to the maximum quantities from each treatment, as described previously^75^. Values not followed by the same letter indicate a significant difference. Nonmetric multidimensional scaling (NMDS) plots of the dissimilarities in Bray-Curtis distance-based community composition among “heavy” and “light” DNA fractions in Control (**D**), NPK (**E**) and OM (**F**) soils incubated with ^13^C-straw, ^12^C-straw or Unamended.

After ultracentrifugation, the bacterial 16S rRNA gene copy numbers across the isotopically fractionated DNA gradients were quantified. The bacterial 16S rRNA gene copy numbers of the “heavy” DNA fractions (buoyant densities ranging from ca. 1.7250 g ml^−1^ to ca. 1.7354 g ml^−1^) from the ^13^C-straw treatments were found higher than those of the ^12^C-straw and those of the Unamended treatments (i.e., at 1.735 g ml^−1^ of buoyant density, increasing from 1.3% to 25.9% for Control, from 3.2% to 22.8% for NPK and from 0.8% to 27.6% for OM, respectively) (*P* < 0.05) (Fig. 2A–C). This implies that some species had assimilated ^13^C-atom derived from ^13^C-straw, which made their appearances in the heavy DNA fractions.

### Shifts in bacterial community composition of isotopically fractionated DNA gradients

Non-metric multidimensional scaling (NMDS) plots revealed the shifts in the bacterial community composition between different samples (Fig. 2D–F), and this was statistically confirmed by permutation tests (Tables S2–S4) based on the amplicon profiling of the bacterial 16S rRNA gene. Significant shifts in the composition were exclusively observed between “heavy” and “light” DNA fractions of the ^13^C-straw, ^12^C-straw and the Unamended treated soils. This indicates that DNA from all samples was successfully fractionated. Furthermore, significant shifts in the community composition of the “heavy” DNA fractions were observed between ^13^C-straw, ^12^C-straw and Unamended treatments. This indicates that some bacterial species that actively assimilated ^13^C from ^13^C-straw were successfully isolated by ultracentrifugation.

### Differences in straw degrading bacterial species among fertilizations

**Figure 3:**
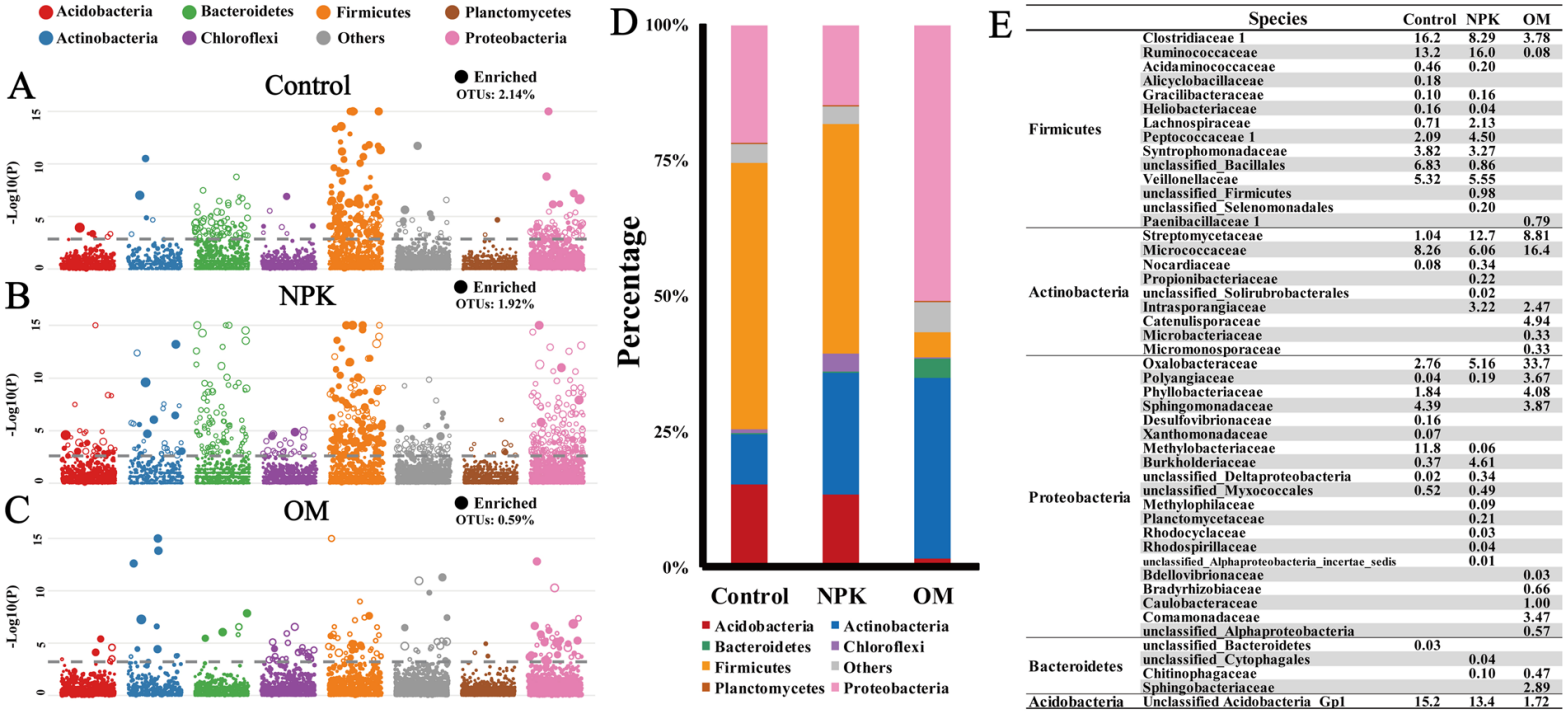
Changes in straw degrading bacterial species among different fertilizations. Solid circles in Manhattan plots represent the positively responding OTUs (defined as the responders) in the “heavy” DNA fractions of ^13^C-straw treatments, compared to ^12^C-straw treatments, in Control (**A**), NPK (**B**) and OM (**C**) soils. The dashed line corresponds to the false discovery rate-corrected *P* value threshold of significance (α = 0.05). The 100% stacked column chart shows the relative abundance of the predominant responders in the “heavy” fraction of ^13^C-straw treatments (Control, NPK and OM) (**D**). The detailed taxonomic information on responders’ community shifts (percentage) among Control, NPK and OM (**E**).

By comparing the relative abundances of community constituents in the “heavy” DNA fractions between ^13^C- and ^12^C-straw treatments, the positive responding OTUs (solid circles above a threshold of significance) in ^13^C-straw treatments were defined as the responders, the putative straw decomposers (Fig. 3A–C). Generally, 2.14%, 1.92% and 0.59% of the total OTUs can be defined as the responders for the Control, NPK and OM soils respectively. In Control soils, the most responders were affiliated with *Firmicutes* (Fig. 3D), accounting for 49.12% of the positive responding sequences, followed by *Proteobacteria* (21.91%), *Acidobacteria* (15.23%) and *Actinobacteria* (9.38%). The predominant responders in NPK soils were also assigned to *Firmicutes* (42.29%), followed by *Actinobacteria* (22.52%), *Proteobacteria* (14.91%) and *Acidobacteria* (13.37%). However, OM fertilization led to increased percentages of the responders belonging to *Proteobacteria* (51.04%), *Actinobacteria* (33.28%) and *Bacteroidetes* (3.36%) (Fig. 3D), while those affiliated to *Acidobacteria* (1.72%) and *Firmicutes* (4.65%) were decreased. At the higher resolution, in Control soils, the responders were classified into *Clostridiaceae* (16.23%), *Ruminococcaceae* (13.22%) and *Veillonellaceae* (5.32%) within *Firmicutes* and *Methylobacteriaceae* (11.75%), *Sphingomonadaceae* (4.39%) and *Oxalobacteraceae* (2.76%) within *Proteobacteria* (Fig. 3E). In NPK soils, the responders were mainly *Clostridiaceae* (8.29%) and *Ruminococcaceae* (16.01%) within *Firmicutes* and *Streptomycetaceae* (12.67%) and *Micrococcaceae* (6.06%) within *Actinobacteria*. In OM soils, the responders became *Oxalobacteraceae* (33.69%) and *Phyllobacteriaceae* (4.08%) within *Proteobacteria* and *Micrococcaceae* (16.40%), *Streptomycetaceae* (8.81%) and *Catenulisporaceae* (4.94%) within *Actinobacteria* and *Sphingobacteriaceae* (2.89%) within *Bacteroidetes*. Pairwise comparison indicated that each soil had some responders in common with other soils whilst some responders were exclusive (Fig. 3E). For example, *Clostridiaceae*, *Ruminococcaceae*, *Streptomycetaceae*, *Micrococcaceae*, *Oxalobacteraceae* and *Polyangiaceae* were commonly observed in all fertilized soils, while *Alicyclobacillaceae* were only detected in Control soils, *Propionibacteriaceae* and *Planctomycetaceae* were only observed in NPK soils, and *Paenibacillaceae*, *Catenulisporaceae*, *Caulobacteraceae*, *Comamonadaceae* and *Sphingobacteraceae* were only for OM soils.

### Patterns of bacterial molecular ecological network among fertilizations

**Figure 4:**
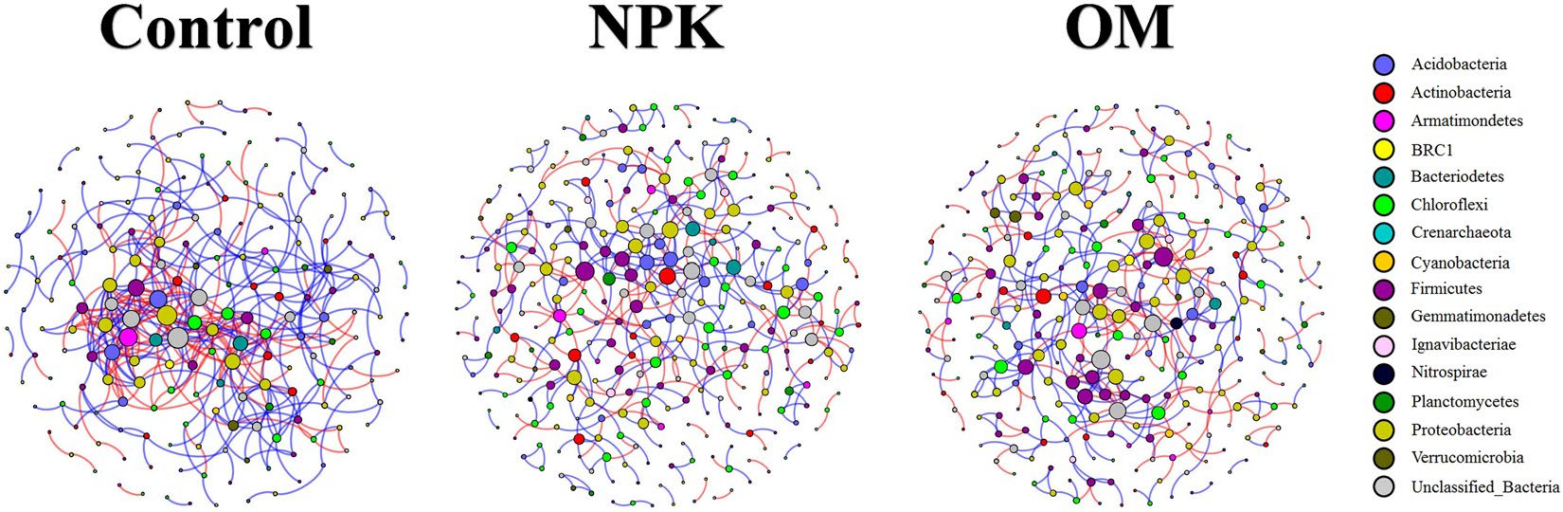
The visualization of the bacterial network associations in Control, NPK and OM soils based on RMT analysis from OTU profiles. Red and blue lines respectively represent negative and position correlations between nodes.

Phylogenetic molecular ecological networks (pMENs) were conducted to determine the influence of different fertilizations on the bacterial interspecies interaction under straw amendment. The topological properties that characterize the complexity of inter-relationships among ecotypes were calculated (Fig. 4 and Table 1). Random networks were generated to test the statistical significance of the network indices among fertilizations (Table S5). Permutation tests indicate that the majority of the network indices were significantly different among Control, NPK and OM soils (*P* < 0.001) (Table S6). Particularly, the values of density, degree of centralization, average degree and transitivity were statistically the highest in the Control soils (*P* < 0.001), while values of modularity, total number of nodes, average path distance and harmonic geodesic distance were significantly higher for NPK and OM soils (*P* < 0.001). NPK (37) and OM (33) soils had the higher module numbers than Control (20) (Fig. S1). These phenomena suggest that fertilization simplified and decentralized the bacterial molecular ecological network. Further eigengene analysis indicated that the hub members of maximum betwennness, affiliated with *Firmicutes* (*Clostridiales*) in Control soils, shifted to those in *Proteobacteria* in the NPK (*Caulobacterales*) and OM (*Myxococcales*) soils (Table 1).

**Table 1:** Topological properties of bacterial molecular ecological networks among different fertilization strategies.

Topological properties	Control	NPK	OM
Density (D)	0.021	0.009	0.010
Centralization of degree (CD)	0.085	0.020	0.024
Centralization of betweenness (CB)	0.141	0.131	0.134
Geodesic efficiency (E)	0.253	0.167	0.158
Centralization of eigenvector centrality (CE)	0.266	0.351	0.356
Average clustering coefficient (avgCC)	0.269	0.261	0.294
Maximal eigenvector centrality	0.294	0.367	0.376
Transitivity (Trans)	0.427	0.328	0.348
Connectedness (Con)	0.612	0.605	0.605
Centralization of stress centrality (CS)	0.788	0.353	0.773
R square of power-law	0.954	0.744	0.788
Efficiency	0.973	0.99	0.989
Harmonic geodesic distance (HD)	3.949	5.991	6.32
Average degree (avgK)	4.219	2.745	2.947
Average path distance (GD)	5.239	7.63	8.836
Maximal degree	21	9	10
Total nodes	201	314	300
Total links	424	431	442
Nodes with max betweenness	Clostridiales	Caulobacterales	Myxococcales

## Discussion

### Taxonomically diverse straw decomposers in paddy soils

Understanding the mechanisms underlying the operation of microbial-driven decomposition of straw is crucial for the understanding of C sequestration and its impact on arable soils’ fertility. Although fungi are mainly involved in decomposition of straw in oxic soils, at anoxic conditions bacterial species are the predominant decomposers, especially in paddy soils^31^. For this reason, only bacterial decomposers were focused in this investigation. The decomposers were found taxonomically diverse and mainly affiliated with the *Firmicutes*, *Actinobacteria*, *Proteobacteria* and *Bacteroidetes* phyla (Fig. 3). This finding is consistent with the previous reports^14,32,33^. Specifically, the strictly anaerobic genus *Clostridium* is a major member of the bacterial community responsible for the degradation of plant residues under anaerobic conditions^13,34,35^. *Lachnospiraceae*, *Ruminococcaceae* and *Veillonellaceae* were also identified as the most abundant and dynamic bacterial species involved in the anoxic breakdown of rice straw^31^. All of these organisms possess large numbers of genes that encode plant cell wall-degrading enzymes that metabolize hemicellulose and/or cellulose^7,36,37^. Due to the similar habitats, these species are also found in animal rumens^7,37,38^. Some aerobic decomposers were also observed, such as *Arthrobacter*, *Streptomyces* and *Bacillus* (Fig. 3)^10,33^. This suggests that aerobes could be still active in degrading straw in flooded paddy soils. There are two possible explanations: 1) there are oxic and/or micro-oxic habitats in paddy soils^39^; 2) these species could be facultative aerobes. In all soils, *Clostridiaceae* and *Ruminococcaceae* within *Firmicutes*, *Streptomycetaceae* and *Micrococcaceae* within *Actinobacteria* and *Oxalobacteraceae* and *Polyangiaceae* within *Proteobacteria* were the common decomposers (Fig. 3E). It is known that *Streptomyces* plays an important role in assimilating complex carbohydrates in soils^40,41^. *Micrococcaceae*^42^ and *Polyangiaceae*^43^ are primary decomposers with well-known cellulolytic activity. This indicates that these straw degrading species could remain unaffected by fertilization and that they could be widely distributed in paddy soils.

Cross feeding is one of the inevitable limitations of DNA-SIP^44^. Prolonged incubation can increase the extent of labeling and inevitably lead to ^13^C spread throughout the community due to cross-feeding (involving metabolic byproducts or dead cells). Thus, cross feeding could cause overestimation of the targeted species. In this investigation, several responders that could not necessarily participate into the degradation of straw could have been labeled possibly due to this limitation; *Rhodospirillaceae*, *Desulfovibrionaceae* and *Methylobacteriaceae* may be some of these organisms. ^13^C-straw degradation could provide with large amounts of ^13^C-labeled short chain fatty acids and then emit ^13^C-CH_4_ as per the most common methanogenic pathway through acetate. These species could assimilate such compounds and were thus identified. We are confident that the cross-feeding effect was kept minimal at this trial due to the short duration of incubation (25 days in this case). Furthermore, DNA-SIP only enables the identification of microorganisms with high-abundance labeling. Other microorganisms that utilize mixed carbon sources or obtain slow growth with low ^13^C labeling but contribute to straw degradation could have been poorly labeled and got underestimated. The incubation condition of microcosm and the gradient centrifugation could have also led to certain biases. It is exemplified that in this study NPK-Unamended soils had the higher buoyant densities compared to NPK-^12^C soils (Fig. 2B).

### Fertilization promotes anoxic straw degradation process

The majority of the straw decomposers observed at the Control soils were affiliated with *Firmicutes*, while decomposers affiliated with *Actinobacteria*, *Acidobacteria* and *Bacteroidetes* were the most significant phyla under NPK fertilization, whilst those within *Proteobacteria*, *Actinobacteria* and *Bacteroidetes* were the predominant under OM (Fig. 3D). It is well known that decomposition processes are conceptually separated by a rapid and a slower phase during early and the later stage respectively^15^. Correspondingly, the organic components of plant residues can be divided into easily degradable fractions, such as long/short chain fatty acids and less-degradable or more persistent to treatment fractions, including cellulose and lignin. *Clostridia* sp. was predominant during the early stage of anoxic straw degradation process in paddy soils^19,20,31,35^. On the contrary, species within *Proteobacteria*, *Bacteroidetes* and *Actinobacteria* are often found to be the active decomposers of the less-degradable or more persistent plant residue components. For example, the isolated members of *Oxalobacteraceae* have high chitinolytic, proteolytic and collagenolytic activities^45^; Species of *Sphingobacteriales*, *Caulobacters* and *Comamons* have a high potential in degrading plant-derived biopolymers^46–48^. For this reason, these decomposers are observed in the later succession of the straw degradation^13^. The presence of a higher number of such decomposers in the fertilized soils, compared to that in the Control soils, suggests that the straw degradation process was accelerated. This finding is also supported by the net mineralization data (Fig. 1C) and is consistent with the previous findings^22^.

### Fertilization influences community composition of straw decomposers

The complex composition of straw mandates the presence and/or the development of a well-organized microbial community for the accomplishment of a multi-stage, anoxic degradation process. Therefore, it is reasonable to believe that there is a link between fertilization and the promotion or screening of straw decomposing microbial communities. Generally, the lower ratios of straw decomposers over the total OTUs were observed for NPK and OM soils in comparison with Control soils (Fig. 3A–C). This implies that fertilization influenced community composition of straw decomposers, which confirmed our first hypothesis. It is well known that bacterial phyla can be classified into the copiotrophic and the oligotrophic, corresponding to the *r*- and *K*-selected categories respectively^9^. *Acidobacteria* are classified as *K*-strategists and β-*Proteobacteria* and *Bacteroidetes* are *r*-strategists. OM fertilized soil has higher soil fertility, which, in turn could favor *r*-strategists. For example, straw has a high C:N ratio and low N content. More N nutrient in environments is beneficial to microorganisms for the degradation of crop residues^49,50^. Fertilization increased soil N-nutrient content (Table S1), which could stimulate *r*-strategy decomposers. Therefore, the relative abundances of *Sphingobacteriaceae* within *Bacteroidetes* and *Comamonadaceae* within β-*Proteobacteria* in straw decomposers were inevitably highest in the OM fertilized soils. On the contrary, Control had relatively lower soil fertility and subsequently limited population of copiotrophic decomposers, which led to a relatively slower degradation process in comparison with the one achieved in fertilized soils. This explains why *Clostridiaceae* I and the oligotrophic *Acidobacteria*, were the predominant straw decomposer in the Control soils during 25-day incubation.

### Fertilization makes the decomposers with high degrading potential pivotal in the consortia

Fertilization also influenced the molecular ecological network of the soil microbiome under straw amendment. The developed networks for the three soils under straw amendments were exclusively nonrandom (Table S5). These deterministic processes render the networks more robust to random disruption^51^. Further pairwise comparisons revealed that higher module numbers and lower degrees of centralization, complexity and transitivity were observed for NPK and OM rather than for Control soils (Tables 1 and S6). Decentralization and simplification of the microbiome is a proposed way to promote ecosystem stability via sufficient avoidance of cascading collapses of the interspecies network in the presence of disturbances. Changes in transitivity, average harmonic geodesic distances and geodesic efficiencies also support the above scenario. Lower transitivity indicates weaker interactions and couplings within the community^52^, hence stronger stability of the co-occurrence network is achieved^53^. The smaller average harmonic geodesic distances and the larger geodesic efficiencies of the Control network indicate that all nodes are closer^54,55^ and fertilization decreases the interspecies collaboration. These phenomena are also attributed to the higher soil trophic level in response to fertilization (Table S1). As streamlining theory predicts, the increase in the competition for resources is always concurrent with the increases in the cell-cell interactions among microbial members^56^. Consistently, based on the first principles of thermodynamics, Großkopf and Soyer^57^ found that when energy resources are limited, microorganisms tend to coexist for survival. Thus, it is postulated that the high complexity in a co-occurrence network due to such interspecies dependency may not favor microbial ecological functions^30^. On the contrary, the simplified biotic co-occurrence network under fertilizations could accelerate the microbial ecological functions. It is corroborated that the module numbers increased from 20 (Control) to 37 and 33 for NPK and OM respectively (Fig. S1). In view of network modules equivalent to potential ecological functions, the presence of more modules under fertilization implies more microbial ecological functions. As microbial function is highly correlated with carbon sequestration^58,59^, it is reasonable to deduce that it is one of mechanisms of fertilization promoting straw degradation (Fig. 1C).

Keystone species are generally crucial to the entire network, and their absence may cause catastrophic changes in the ecosystem^60^. The species affiliated with the *Proteobacteria* phylum became more important in the microbial network of OM soils (Fig. S2). The high betweenness node can serve as an important broker^27^. It was found that the species with the maximum betweenness were also shifted from *Clostridiales* in *Firmicutes* for the Control to *Caulobacterales* in *Proteobacteria* for the NPK and *Myxococcales* in *Proteobacteria* for the OM (Table 1). The species of *Caulobacteriales*^47^ and *Myxococcales*^61^ had a high potential for degrading polymerised organic matter. This suggests that fertilization made the decomposers with high degrading potential pivotal in the consortium. These results confirmed our second hypothesis that fertilization influences the synergistic interactions between species, especially the keystone degrading species. Their increased significance in the ecological module is speculated to function as vanguard accelerating anoxic straw degradation in paddy soils. The speculation is the co-metabolism of microorganisms, one of the mechanisms of the priming effect described by Fontaine *et al*.^62^. In fertile soils, those decomposers specializing in the consumption of polymerised soil organic matters can readily produce extracellular depolymerising enzymes. Due to the similar polymerised structures, these enzymes are efficient for the degradation of straw. Furthermore, this process is speculated to produce catabolites that help other decomposers to better execute straw degradation. Thus, a greater extent of shifts in the community of decomposers was observed in OM soils with the higher soil organic matter content, compared to NPK soils.

Straw degradation necessitates numerous metabolic steps and subsequently a succession of intimate microbial consortia. In this investigation, we solely focused on the bacterial decomposers developed at an early stage of rice straw degradation. A more persistent to degradation fraction of rice straw remained unhydrolyzed. Prolonged incubation could have triggered the formation of species-richer communities with even more complex symbiotic interactions that could have further convert and ferment straw substrates into methane.

## Materials and Methods

### Cultivation of high-abundance ^13^C-labeled rice straw

One month-old rice seedlings (*Oryza sativa* L.) were transplanted to a gas-tight growth chamber and exposed to ^13^C-CO_2_-enriched atmosphere for 60 days. The chamber was set-up according to Bei *et al*.^63^. Three sub-controlling systems incorporated in this unit: a plant growth chamber, a temperature and CO_2_ concentration controlling system and a ^13^C-CO_2_ generator. The automatic control system was based on a data-logger (CR10x, Campbell Scientific, Logan, Utah, USA) which performed real-time measurements of temperature and CO_2_ concentration via a temperature sensor and infrared CO_2_ analyzer respectively. Sampling intervals were 30s and data were recorded every 60s for temperature and CO_2_ concentration. The ^13^CO_2_ was generated from the reaction between H_2_SO_4_ and Na_2_^13^CO_2_ (^13^C 99%: Cambridge Isotope Laboratories, Andover, MA, USA). The CO_2_ (^13^C-CO_2_ (>99.99%)) generator was activated at chamber concentrations <350 ppm. Conversely, when CO_2_ concentration in the chamber was >410 ppm, ^13^CO_2_ generation was stopped and a switch diverted excess gas flow through CO_2_ traps (NaOH solution) to absorb redundant CO_2_. The chamber was disconnected after 60 days of labeling. All aboveground parts of rice were separated, dried, chopped and mixed before their application to the soil microcosms. The ratio of ^13^C to ΣC (ΣC: ^13^C + ^12^C atoms) of rice straw was ca. 70%. The natural rice straw was prepared by routine rice cultivation in the greenhouse, and the ratio of ^13^C to ΣC was ca. 1.08%.

### Laboratory ^13^C-labeled rice straw degradation microcosms

Paddy soils exposed to a 26-years application of different fertilization regimes (balanced chemical fertilizers (termed NPK afterward), organic amendments (OM) or without fertilization (Control)) were collected from Yingtan red soil ecological experimental station, Jiangxi Province, China (28°15′N, 116°55′ E). The chemical properties of the soils are presented on Table S1. The soils were prepared as inocula for microcosm activity/biodegradation essays.

For the microcosm assays, 10 g of soil was added to serum bottles (120 ml, 10 cm in height) and preincubated for 3 days in a dark chamber at 27 °C. 0.1 g ^13^C-labeled rice straw with pieces of approximately 0.5 cm was then added to each serum bottle (^13^C-straw treatment). The soil moisture was adjusted to 60% of water holding capacity. Before incubation, the headspace of the serum bottle was vacuumed and flushed with N_2_ gas (99.9%). The serum bottles were then anoxically incubated in a dark chamber at 27 °C and remained sealed for 25 days. Two parallel treatments were conducted for comparison. One treatment was amended with natural rice straw (^12^C-straw), whilst the other only contained soils without rice straw amendment (Unamended). Each treatment was prepared in 8 replicates (total of 72 microcosms). Gas samples were collected from the headspace of microcosms at days 5, 10, 15, 20 and 25 using a gas-tight syringe. The concentrations of CO_2_ and CH_4_ were analyzed by gas chromatography with ECD (Agilent 7890A, Agilent Technologies). Abundances of ^13^C-CO_2_ were analyzed by GC-IRMS using a pre-concentration unit (Thermo Finnigan Delta C + and Precon, Thermo Finnigan, Bremen, Germany). On the day after final gas sampling, the soils from each serum bottle were collected and stored at −40 °C for DNA extraction and further molecular analysis.

### DNA extraction

DNA from 0.5 g of soil from each microcosm was extracted using the ‘FastDNA^®^ SPIN kit for soil’ according to the manufacturer’s instructions (MP Biomedicals, Santa Ana, CA). The extracted DNA was eluted in 50 μl of TE buffer, quantified by Nanodrop 2000 (Thermo, USA) and stored at −20 °C until further use.

### Isopycnic centrifugation and gradient fractionation

Random 5 DNA replicates from each treatment were conducted to stable isotope probing fractionation, according to Jia and Conrad^64^ and Neufeld *et al*.^65^. “Heavy” and “light” DNA were separated by density gradient ultracentrifugation using CsCl. The gradient mixture consisted of mixing 3.95 ml of CsCl solution and transferred to 4.9 ml tubes. Density gradient centrifugation was performed in at 177,000 *g* for 44 h at 20 °C. The centrifuged gradients were fractionated from bottom to top into 15 equal fractions. DNA was precipitated with polyethylene glycol 6000 and dissolved in 30 μl of TE buffer after washing by 70% ethanol.

### Real-time quantitative PCR of bacterial 16S rRNA gene

The copy numbers of the bacterial 16S rRNA gene fragments in each DNA fractions were quantified by real-time quantitative PCR (qPCR) using the 519 F/907R primer set. The targeted gene copy number was quantified by qPCR analysis using C1000^Tm^ Thermal Cycler equipped with a CFX96^Tm^ Real-Time system (Bio-Rad, USA). Standard curves were obtained using 10-fold serial dilutions of the linear *Escherichia coli*-derived vector plasmid pMD18-T (TaKaRa, Japan) containing a cloned target gene, using 10^2^ to 10^7^ gene copies μl^−1^. The reactions were performed at a C1000^TM^ Thermal Cycler equipped with a CFX96^TM^ Real-Time System (Bio-Rad, USA). The 25-μl reaction mixture contained 12.5 μl of SYBR^®^ Premix Ex Taq^TM^ (TaKaRa, Japan), 0.5 μM of each primer, 200 ng bovine serum albumin μl^−1^, and 1.0 μl of template containing approximately 2–9 ng DNA. Blanks were run with water as template extract. The qPCR program applied included the following steps: 94 °C for 5 minutes, followed by 35 cycles of 94 °C for 30s, 55 °C for 30s and 72 °C for 60s, and extension and signal reading. The specificity of the amplification products was confirmed by melting curve analysis, and the expected size of the amplified fragments was checked using a 1.5% agarose gel. qPCR was performed in triplicate and amplification efficiencies of 97.4 to 104% were obtained with *R*^2^ values of 0.966 to 0.977.

### Preparation of the amplicon libraries for high-throughput sequencing

Bacterial communities from i) the ^12^C-straw treatment without ultracentrifugation and ii) those in “light” and “heavy” DNA fractions of ^13^C- and ^12^C- straws and Unamended microcosms in the SIP experiment were analyzed by high-throughput sequencing. Each DNA template was amplified using the 515F and 907R primer set to approximately 400 bp of bacterial 16S rRNA gene (V4-V6 fragments^66^). Briefly, the oligonucleotide sequences included a 5-bp barcode fused to the forward primer as follows: barcode + forward primer. PCR was carried out in 50 µl reaction mixtures with the following components: 4 µl (initial: 2.5 mM each) of deoxynucleoside triphosphates, 2 µl (initial: 10 mM each) of forward and reverse primers, 2 U of *Taq* DNA polymerase with 0.4 µl (TaKaRa, Japan), and 1 µl of template containing approximately 50 ng of genomic DNA. Thirty-five cycles (95 °C for 45s, 56 °C for 45s, and 72 °C for 60s) were performed with a final extension at 72 °C for 7 min. Triplicate reaction mixtures per sample were pooled, purified using the QIAquick PCR Purification kit (QIAGEN) and quantified using a NanoDrop ND-1000 (Thermo, USA). The barcoded PCR products from all samples were normalized in equimolar amounts before sequencing by using a MiSeq Reagent Kit v2 (2 × 250 cycles) following the manufacturer’s protocols. The sequences were deposited in the NCBI SRA database (accession no. SRP128685).

### Processing of high-throughput sequencing data

Raw sequencing data were assembled with FLASH^67^ and processed with the UPARSE algorithm^68^. Thereinto, primers were trimmed with ‘cutadapt’ (Version 1.9.2)^69^. Then sequences with average quality score below 25 and length less than 300 bp were discarded and chimera was filtered by UPARSE. Operational taxonomic units (OTUs) were delineated using a 97% similarity threshold, and taxonomy was determined using the RDP classifier for bacteria^70^. “Heavy” and “light” DNA fractions gave 7,611,782 sequences of bacterial 16S rRNA gene, ranging from 6,830 to 52,978 sequences per sample, median value of 25,747 sequences per sample. DNAs from ^12^C-straw treatment without fractionation gave 670,133 sequences of bacterial 16S rRNA gene, ranging from 8,171 to 50,902 sequences per sample, median value of 23,317 sequences per sample. To ensure even depth of sampling for diversity calculations and diversity metrics a subset of 6,500 and 8,000 sequences per sample was selected respectively for fractionated and non-fractionated DNA samples. The Bray-Curtis distance was calculated for the comparisons of the taxonomical community composition, results were visualized using non-metric multidimensional scaling (NMDS) plots.

### Identification of ecotypes metabolically actively assimilating rice straw

For the identification of the straw decomposers Manhattan plots were estimated via edger and dplyr packages and plotted with the gplots package in R (Version 3.1.2), according to the protocol of Zgadzaj *et al*.^71^. The putative straw decomposers were defined as the positively responding OTUs above a threshold of significance (false discovery rate-corrected *P* values, α = 0.05) in the “heavy” DNA fractions of ^13^C-straw amended microcosms in comparison with the corresponding DNA fractions of ^12^C-straw microcosms.

### Molecular ecological network analysis

Changes in the phylogenetic molecular ecological networks (pMENs) of the ^12^C-straw treatment without fractionation among the three fertilizations were evaluated using the random matrix theory (RMT)-based network approach^72^. The pMEN construction and analyses were performed using a pipeline written in Java and Perl scripts (http://129.15.40.240/mena/login.cgi^27^). The network graphs were visualized by Gephi software. Indexes, such as density, average centralization of degree, transitivity, average degree, average path distance and geodesic efficiency of pMEN were used to evaluate the changes in biotic interaction within community in response to fertilizations. To decipher the importance of hub species in co-occurrence network, species were sorted into four subcategories: peripherals, connectors, module hubs, and network hubs^73^. Identification of the keystone species in the network was conducted via eigengene analysis using maximum Betweenness as a point of reference. Betweenness is used to describe the ratio of paths that pass through the *i*^th^ node (${B}_{i}={\sum }_{jk}\frac{{\rm{\sigma }}\,(j,\,i,\,k)}{{\rm{\sigma }}\,(j,\,k)}$, σ (*j*, *k*) is the total number of shortest paths between *j* and *k*.)^27^.

### Statistical analysis

Statistical analysis was carried out using the IBM Statistical Product and Service Solutions (SPSS) Statistics for Windows (Version 13). The data were expressed as the means with standard deviation (SD); different letters indicate significant differences between different samples. Mean separation among fertilizations was conducted based on Tukey’s multiple range test, following the tests of assumptions of normal distribution, homogeneity of variance and ANOVA. Permutational multivariate analysis of variance (PERMANOVA)^74^ was conducted to test the statistically significant differences of community composition between “light” and “heavy” DNA fractions of ^13^C- or ^12^C-straw treatments or Unamended treatment, using R software (the vegan package, Version 3.1.2).

## Electronic supplementary material

Supplementary information
