# Supplementary material for: Fertilization shapes a well-organized community of bacterial decomposers for accelerated paddy straw degradation
Source: Sci Rep. 2018 May 22;8:7981. doi: 10.1038/s41598-018-26375-8 (PMC5964224; doi:10.1038/s41598-018-26375-8)

**Supplementary information**

**Title:** Fertilization shapes a well-organized community of bacterial decomposers for accelerated paddy straw degradation

**List of authors:** Yushan Zhan^a^, Wenjing Liu^a^, Yuanyuan Bao^a^, Jianwei Zhang^a^, Evangelos Petropoulos^b^, Zhongpei Li^a^, Xiangui Lin^a^, Youzhi Feng^a,*^

Author affiliations:

^a^State Key Laboratory of Soil and Sustainable Agriculture, Institute of Soil Science, Chinese Academy of Sciences, Nanjing, 210008, PR China

^b^School of Civil Engineering and Geosciences, Newcastle University, Newcastle upon Tyne NE1 7RU, UK

**Corresponding author^*^**

Youzhi Feng, Tel: +086-025-86881367; Fax: +086-025-86881000

E-mail: [yzfeng@issas.ac.cn](mailto:yzfeng@issas.ac.cn)

**Table S1.** The chemical information of fertilized soils in Yingtan ecological experimental station

| **Treatments** | **Total N**  **(g/kg)** | **AN**  **(mg/kg)** | **SOM**  **(%)** | **AP**  **(mg/kg)** | **AK**  **(mg/kg)** | **Total K**  **(%)** | **Total P (mg/kg)** | **pH** |
| --- | --- | --- | --- | --- | --- | --- | --- | --- |
| Control | 0.83±0.06a | 62.83±7.24a | 1.37±0.14a | 0.28±0.12a | 126.67±17.47b | 1.68±0.05a | 304.33±13.05a | 5.25±0.09a |
| NPK | 1.13±0.13b | 83.87±9.09b | 1.84±0.22b | 2.56±0.44b | 70±7.21a | 1.57±0.07a | 534.33±56.98b | 5.36±0.06a |
| OM | 1.24±0.08b | 101.53±8.73c | 1.99±0.18b | 5.84±0.66c | 106±2.00b | 1.57±0.11a | 714.33±21.50c | 5.33±0.21a |

**Table S2.** PERMANOVA showing the significant Bray-Curtis distance-based dissimilarities of bacterial community composition between “heavy” and “light” DNA fractions in Control samples treated with ^13^C-, ^12^C-straw or Unamended, as well as those in “heavy” DNA fractions between ^13^C- and ^12^C-straw and between ^13^C-straw and Unamended.

| Pairwise comparison | F.Model | *R*^2^ | p.value | p.adjusted |
| --- | --- | --- | --- | --- |
| ^13^C_Heavy vs ^13^C_Light | 6.343 | 0.209 | 0.001 | 0.002 |
| ^12^C_Heavy vs ^12^C_Light | 9.306 | 0.271 | 0.001 | 0.002 |
| Unamended_Heavy vs Unamended_Light | 3.538 | 0.124 | 0.015 | 0.016 |
| ^13^C_Heavy vs ^12^C_Heavy | 10.99 | 0.250 | 0.001 | 0.002 |
| ^13^C_Heavy vs Unamended_Heavy | 10.64 | 0.244 | 0.001 | 0.002 |

**Table S3.** PERMANOVA showing the significant Bray-Curtis distance-based dissimilarities of bacterial community composition between “heavy” and “light” DNA fractions in NPK samples treated with ^13^C-, ^12^C-straw or Unamended, as well as those in “heavy” DNA fractions between ^13^C- and ^12^C-straw and between ^13^C-straw and Unamended.

| Pairwise comparison | F.Model | *R*^2^ | p.value | p.adjusted |
| --- | --- | --- | --- | --- |
| ^13^C_Heavy vs ^13^C_Light | 11.60 | 0.317 | 0.001 | 0.001 |
| ^12^C_Heavy vs ^12^C_Light | 11.54 | 0.316 | 0.001 | 0.001 |
| Unamended_Heavy vs Unamended_Light | 13.58 | 0.361 | 0.001 | 0.001 |
| ^13^C_Heavy vs ^12^C_Heavy | 9.255 | 0.214 | 0.001 | 0.001 |
| ^13^C_Heavy vs Unamended_Heavy | 19.74 | 0.374 | 0.001 | 0.001 |

**Table S4.** PERMANOVA showing the significant Bray-Curtis distance-based dissimilarities of bacterial community composition between “heavy” and “light” DNA fractions in OM samples treated with ^13^C-, ^12^C-straw or Unamended, as well as those in “heavy” DNA fractions between ^13^C- and ^12^C-straw and between ^13^C-straw and Unamended.

| Pairwise comparison | F.Model | *R^2^* | p.value | p.adjusted |
| --- | --- | --- | --- | --- |
| ^13^C_Heavy vs ^13^C_Light | 6.104 | 0.127 | 0.001 | 0.001 |
| ^12^C_Heavy vs ^12^C_Light | 11.82 | 0.330 | 0.001 | 0.001 |
| Unamended_Heavy vs Unamended_Light | 16.71 | 0.401 | 0.001 | 0.001 |
| ^13^C_Heavy vs ^12^C_Heavy | 4.826 | 0.097 | 0.001 | 0.001 |
| ^13^C_Heavy vs Unamended_Heavy | 8.839 | 0.164 | 0.001 | 0.001 |

**Table S5.** Topological properties of the empirical molecular ecological networks (MENs) of microbial communities and their associated random MENs

| **Network Indexes** | **Control** | | **NPK** | | **OM** | |
| --- | --- | --- | --- | --- | --- | --- |
|  | **Empirical** | **Random** | **Empirical** | **Random** | **Empirical** | **Empirical** |
|  | **Network** | **Network** | **Network** | **Network** | **Network** | **Network** |
|  | **Indexes** | **Indexes** | **Indexes** | **Indexes** | **Indexes** | **Indexes** |
| Average clustering coefficient (avgCC) | 0.269 | 0.044 +/- 0.009 | 0.261 | 0.007 +/- 0.005 | 0.294 | 0.009 +/- 0.005 |
| Average path distance (GD) | 5.239 | 3.584 +/- 0.069 | 7.630 | 5.687 +/- 0.091 | 8.836 | 5.198 +/- 0.107 |
| Geodesic efficiency (E) | 0.253 | 0.315 +/- 0.004 | 0.167 | 0.202 +/- 0.003 | 0.158 | 0.220 +/- 0.003 |
| Harmonic geodesic distance (HD) | 3.949 | 3.173 +/- 0.045 | 5.991 | 4.942 +/- 0.061 | 6.320 | 4.540 +/- 0.070 |
| Centralization of degree (CD) | 0.085 | 0.085 +/- 0.000 | 0.020 | 0.020 +/- 0.000 | 0.024 | 0.024 +/- 0.000 |
| Centralization of betweenness (CB) | 0.141 | 0.111 +/- 0.016 | 0.131 | 0.095 +/- 0.017 | 0.134 | 0.091 +/- 0.015 |
| Centralization of stress centrality (CS) | 0.788 | 0.372 +/- 0.054 | 0.353 | 0.184 +/- 0.034 | 0.773 | 0.200 +/- 0.039 |
| Centralization of eigenvector centrality (CE) | 0.266 | 0.234 +/- 0.021 | 0.351 | 0.241 +/- 0.041 | 0.356 | 0.236 +/- 0.034 |
| Density (D) | 0.021 | 0.021 +/- 0.000 | 0.009 | 0.009 +/- 0.000 | 0.010 | 0.010 +/- 0.000 |
| Transitivity (Trans) | 0.427 | 0.069 +/- 0.007 | 0.328 | 0.011 +/- 0.005 | 0.348 | 0.013 +/- 0.006 |
| Connectedness (Con) | 0.612 | 0.942 +/- 0.031 | 0.605 | 0.916 +/- 0.026 | 0.605 | 0.925 +/- 0.031 |
| Modularity (fast_greedy) | 0.630 | 0.453 +/- 0.007 | 0.841 | 0.668 +/- 0.008 | 0.820 | 0.630 +/- 0.008 |

**Table S6.** T-test about the significance of differences of topological index among three fertilized soil samples

| **T-test** | **Control vs NPK** | **Control vs OM** | **NPK vs OM** |
| --- | --- | --- | --- |
| Average clustering coefficient (avgCC) | 0.0010 | 0.0001 | 0.0052 |
| Average path distance (GD) | 0.0001 | 0.0001 | 0.0001 |
| Geodesic efficiency (E) | 0.0001 | 0.0001 | 0.0001 |
| Harmonic geodesic distance (HD) | 0.0001 | 0.0001 | 0.0001 |
| Centralization of degree (CD) | —— | —— | —— |
| Centralization of betweenness (CB) | 0.0001 | 0.0001 | 0.0792 |
| Centralization of stress centrality (CS) | 0.0001 | 0.0001 | 0.0023 |
| Centralization of eigenvector centrality (CE) | 0.1302 | 0.6173 | 0.3490 |
| Density (D) | —— | —— | —— |
| Transitivity (Trans) | 0.0001 | 0.0001 | 0.0112 |
| Connectedness (Con) | 0.0001 | 0.0001 | 0.0272 |
| Modularity (fast_greedy) | 0.0001 | 0.0001 | 0.0001 |

**Figure S1.** The modules of bacterial network in Control, NPK and OM soils

**
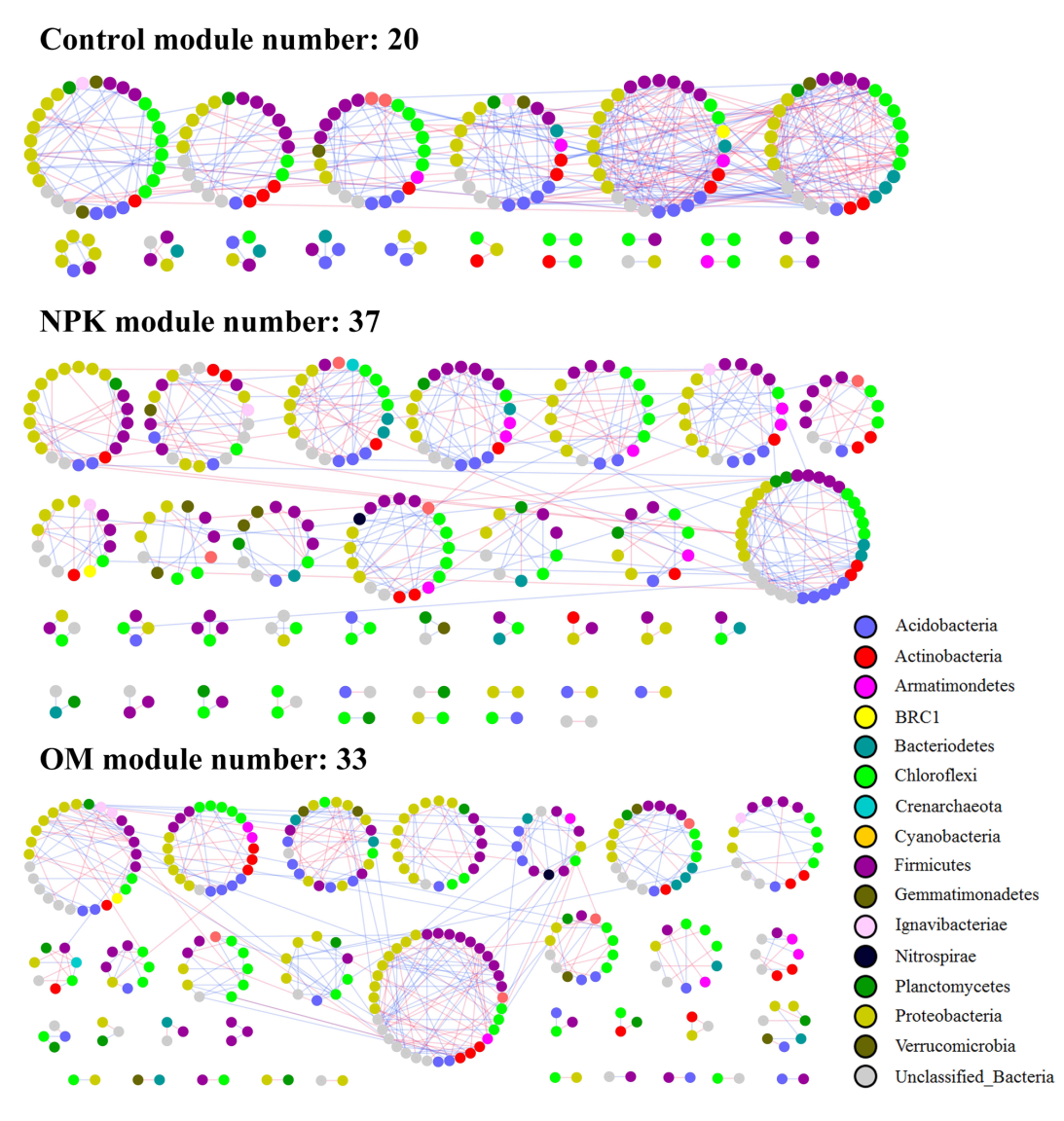
**

**Figure S2.** Zi-Pi plot showing the distribution of OTUs based on their topological roles from three fertilized soils


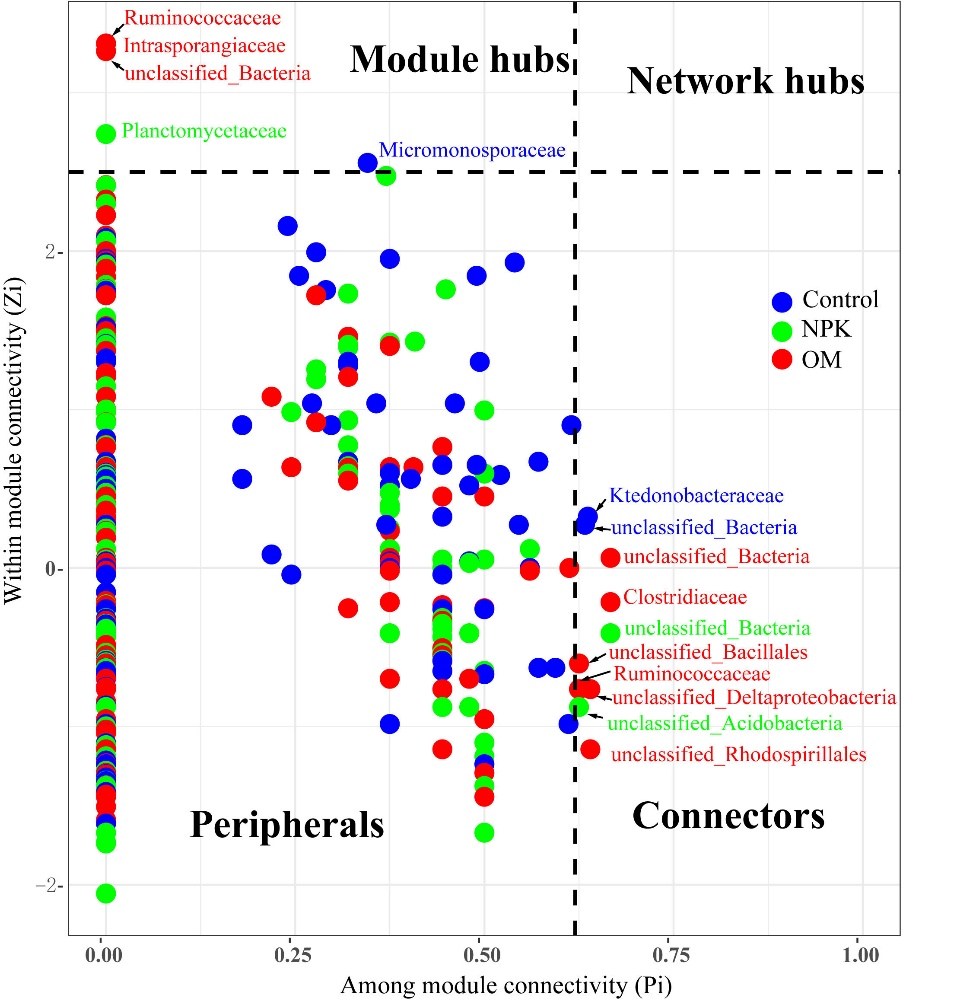

Supplement: Supplementary file 1 — Supplementary information [file 41598_2018_26375_MOESM1_ESM.docx]
